# Addition of Mechanical Lithotripsy to Endoscopic Papillary Large Balloon Dilation in Patients with Difficult Common Bile Duct Stones: A Retrospective Single-Center Study

**DOI:** 10.1089/lap.2023.0274

**Published:** 2023-12-04

**Authors:** Hidehiro Kamezaki, Terunao Iwanaga, Mamoru Tokunaga, Takahiro Maeda, Jun-ichi Senoo, Hiroshi Ohyama, Naoya Kato

**Affiliations:** ^1^ Department of Gastroenterology, Eastern Chiba Medical Center, Togane City, Japan.; ^2^ Department of Gastroenterology, Graduate School of Medicine, Chiba University, Chiba City, Japan.

**Keywords:** mechanical lithotripsy, endoscopic papillary large balloon dilation, common bile duct stones, choledocholithiasis, endoscopic retrograde cholangiopancreatography

## Abstract

**Introduction:**

This study aimed to compare the treatment outcomes of endoscopic papillary large-balloon dilation (EPLBD) with and without mechanical lithotripsy (ML) in removing difficult common bile duct stones.

**Methods:**

Patients with difficult common bile duct stones treated with EPLBD, with or without ML, at the Eastern Chiba Medical Center between April 2014 and March 2020, were retrospectively evaluated. The rates of cumulative recurrence and complications were compared between the two groups.

**Results:**

Overall, 122 patients (*n* = 44, EPLBD + ML and *n* = 78, EPLBD) treated by 12 gastroenterologists were included. Patients in the EPLBD + ML group were older (85 years versus 81.5 years) and had larger maximum stone diameter (15 mm versus 12.5 mm) than those in the EPLBD group. Compared with the EPLBD group, the EPLBD + ML group required more endoscopic retrograde cholangiopancreatography (ERCP) procedures (≥2) (86% versus 67%) and longer total ERCP time after reaching the papilla (78.5 minutes versus 25 minutes). Complication rates were not significantly different (9.1% versus 12.8%); however, the cumulative recurrence rates were higher in the EPLBD + ML group than in the EPLBD group (69.4% versus 23.5% at 4 years).

**Conclusion:**

Although there were no differences in complication rates, the long-term recurrence rate was higher in the EPLBD + ML group than in the EPLBD group. This study emphasizes the added burden imposed by performing ML during ERCP and suggests that by appropriate case selection, it is possible to treat cases of difficult biliary stones using EPLBD without ML.

## Introduction

Endoscopic sphincterotomy (EST) is the standard ampullary intervention for the endoscopic removal of common bile duct stones. Approximately 80%–90% of common bile duct stones can be removed using either a basket or a balloon catheter after EST.^1^ In cases wherein common bile duct stones are difficult to remove using a basket or a balloon catheter, endoscopists often rely on mechanical lithotripsy (ML) or endoscopic papillary large-balloon dilation (EPLBD).

Endoscopic papillary balloon dilation was initially introduced as an alternative to EST to treat 4–10-mm (small to moderate) bile duct stones but was subsequently shown to be also effective in removing large (>10 mm) and/or multiple stones when employed after limited or small EST.^2^ Currently, the indications have expanded, and EPLBD is used effectively in cases with giant stones and often without requiring ML.^3,4^

In a previous study that compared EPLBD and ML for the treatment of difficult common bile duct stones,^5^ EPLBD was observed to reduce treatment duration, increase therapeutic efficacy, and decrease long-term recurrence without increasing the risk of adverse events. Because EPLBD aims to widen the papillary aperture, and ML reduces gallstone size, it can be envisaged that combining these two techniques could have a synergistic effect and increase the extraction rate of large and difficult common bile duct stones. However, using ML may leave debris behind and increase the risk of recurrence. Therefore, it may be preferable to achieve completing treatment with EPLBD alone.

Although the reduced use of ML has been reported in studies using EPLBD,^3,4^ to the best of our knowledge, no studies have directly compared EPLBD and EPLBD + ML in treating common bile duct stones. Therefore, this study aimed to compare treatment outcomes between EPLBD and EPLBD + ML.

## Materials and Methods

### Participants

A retrospective observational study was conducted over a 6-year period (April 2014 to March 2020) at the Eastern Chiba Medical Center. Cases of common bile duct stones where endoscopic retrograde cholangiopancreatography (ERCP) was performed but stone removal with a basket or balloon catheter was difficult and treatment was ultimately performed with EPLBD with or without ML were included in the study. Patients with malignant biliary strictures, those treated with ML alone, and those with incomplete treatment were excluded. Ethical approval was obtained from the ethics committee of Eastern Chiba Medical Center (#41). The requirement for informed consent was waived due to the retrospective nature of the study.

### Endoscopic methods

All procedures were performed by a board-certified fellow of the Japan Gastroenterological Endoscopy Society or by a trainee physician under direct supervision. Midazolam, hydroxyzine, and pentazine were administered for periprocedural sedation and analgesia. After ERCP and EST when needed, stone removal was attempted using a basket or balloon catheter, but proved difficult and unsuccessful. EPLBD was then performed to dilate and disrupt the papillary sphincter, with or without ML, based on the treating clinician's discretion, as previously described.^5,6^

At completion of treatment, complete duct clearance was confirmed by repeated balloon/basket trawls and a balloon occlusion cholangiogram. Patients commenced oral feeding on the first postoperative day if there were no concerns. Patient follow-up and imaging schedules were based on the clinician's discretion.

The following equipment was used for the EPLBD and ML procedures: Extraction Balloon Catheter Plus (Zeon Medical, Inc., Tokyo, Japan) and CRE Pro Wireguided Balloon Dilatation Catheter (Boston Scientific Corp., Marlborough, MA, USA); FlowerBasketV and TetraCatchV Stone Retrieval Baskets (Olympus, Tokyo, Japan); Visiglide and Visiglide 2 Guidewires (Olympus); JF-260V/TJF-260V Duodenoscopes (Olympus); and LithoCrushV Mechanical Lithotripter (Olympus).

### Outcome measures and data collection

Data on patient background, treatment details, intraoperative and postoperative complications, and recurrence were extracted from the medical charts. The recurrence rate of common bile duct stones was the primary outcome measure, and the rate of complications was the secondary outcome measure for the study. Recurrence was defined based on the onset of symptoms of cholangitis or evidence of choledocholithiasis on follow-up imaging in asymptomatic patients.

### Statistical analysis

Patient background, treatment details, and complications were compared between the two groups using the Pearson chi-square test for categorical variables and the Mann–Whitney *U* test for continuous variables. Moreover, cumulative recurrence rates were compared using Kaplan–Meier analysis and log-rank tests. All data were analyzed using SPSS Statistics software (version 24; IBM Japan, Tokyo, Japan), and the significance level was set at *P* < .05.

## Results

### Participants

**FIG. 1.**
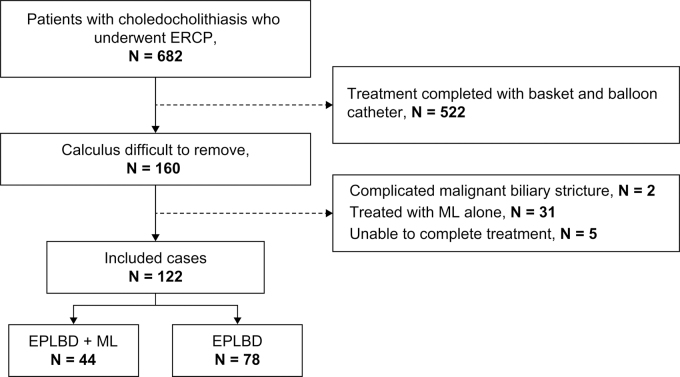
STROBE diagram showing the patients included in the two study groups.

ERCP was performed in 682 patients with common bile duct stones. Of these, 522 patients completed treatment with a basket or balloon catheter, and 160 patients experienced difficulties in stone removal. After excluding 2 patients with a malignant biliary stricture, 31 patients who were treated with ML alone, and 5 patients who did not complete treatment, 122 patients treated by 12 gastroenterologists whose treatment involved EPLBD were included in the study (Fig. 1). Among them, 44 were treated with EPLBD + ML and 78 were treated with EPLBD only. The median follow-up duration was 31.5 days (range, 2 days–4.2 years) in the EPLBD + ML group and 104.5 days (range, 2 days–6 years) in the EPLBD group.

### Patient characteristics

The patient demographics and general background characteristics are given in Table 1. Patients in the EPLBD + ML group were significantly older (median age, 85 years) than those in the EPLBD group (median, 81.5 years; *P* = .028). No significant differences were observed in other patient characteristics (Table 1).

**Table 1.** Characteristics of Patients Treated with Endoscopic Papillary Large-Balloon Dilation+Mechanical Lithotripsy and Endoscopic Papillary Large Balloon Dilation

	EPLBD + ML (*n* = 44)	EPLBD (*n* = 78)	*P*
Median age (minimum–maximum) (years)	85 (65–97)	81.5 (52–99)	.028^*^
Gender (female, %)	59	53	.487
Cholangitis—none: mild: moderate: severe (%)	27:20:43:9	19:22:42:17	.570
Post-EST papilla, *n* (%)	7 (16)	10 (13)	.636
Postoperative reconstructed stomach type, *n*	4 (B-1, B-1, B-2, R-Y)	1 (B-2)	.107
Antithrombotic therapy, *n* (%)	10 (23)	25 (32)	.274
Hypertension, *n* (%)	26 (59)	40 (51)	.406
Diabetes, *n* (%)	10 (23)	19 (24)	.839
Heart disease, *n* (%)	13 (30)	18 (23)	.431
Cerebrovascular disease, *n* (%)	7 (16)	17 (22)	.432
eGFR <30, *n* (%)	2 (5)	7 (9)	.591
Cirrhosis, *n* (%)	0 (0)	2 (3)	.743
Parapapillary diverticulum, *n* (%)	27 (61)	42 (54)	.421
Postcholecystectomy, *n* (%)	9 (20)	9 (12)	.182

^*^ Indicate significance at *P* < .05.

eGFR, estimated glomerular filtration rate; EPLBD, endoscopic papillary large-balloon dilation; EST, endoscopic sphincterotomy; ML, mechanical lithotripsy.

### Endoscopic and intraoperative characteristics

Table 2 summarizes and compares the endoscopic and intraoperative characteristics of the patients in the two groups. The maximum stone diameter was significantly larger in the EPLBD + ML group (15 mm) than in the EPLBD group (12.5 mm, *P* < .001), and most patients in both groups had three or more stones. In addition, the two groups had no significant differences in lower bile duct diameter (median, 13–14 mm) or balloon diameter (median, 12 mm). The balloon diameter of EPLBD is generally considered to be 12 mm or more; however, in this study, in 47 of 78 patients treated with EPLBD, a balloon diameter of <12 mm was used.

**Table 2.** Endoscopic and Intraoperative Characteristics of the Patients Treated with Endoscopic Papillary Large-Balloon Dilation + Mechanical Lithotripsy and Endoscopic Papillary Large-Balloon Dilation

	EPLBD + ML (*N* = 44)	EPLBD (*N* = 78)	*P*
Median lower bile duct diameter (minimum–maximum)	14 (10–19) mm	13 (8–24) mm	.181
Median maximum stone diameter (minimum–maximum)	15 (11–25) mm	12.5 (8–21) mm	<.001^*^
No. of stones, *n* (%)			.416
1 Stone	10 (23)	16 (21)	
2 Stones	4 (9)	14 (18)	
≥3 Stones	30 (68)	48 (62)	
No EST, *n* (%)	3 (7)	2 (3)	.508
Median balloon diameter (minimum–maximum)	12 (10–17) mm	12 (9–18)	.052
Total number of ERCPs, *n* (%)			.018^*^
1 Time	6 (14)	26 (33)	
≥2 Times	38 (86)	52 (67)	
Total ERCP time (after reaching papilla), median (minimum–maximum)	78.5 (16–176) minutes	25 (10–153) minutes	<.001^*^
Post-ERCP cholecystectomy	1/35 (3%)	11/69 (16%)	.099

^*^ Indicate significance at *P* < .05.

EPLBD, endoscopic papillary large-balloon dilation; ERCP, endoscopic retrograde cholangiopancreatography; EST, endoscopic sphincterotomy; ML, mechanical lithotripsy.

The number of patients who required two or more ERCP procedures was significantly higher in the EPLBD + ML group (86%) than in the EPLBD group (67%, *P* = .018). In addition, the total ERCP time after reaching the papilla was significantly longer in the EPLBD + ML group (median, 78.5 minutes) than in the EPLBD group (median, 25 minutes; *P* < .001). Post-ERCP cholecystectomy was performed in 3% of the patients in the EPLBD + ML group and 16% of those in the EPLBD group; however, the differences were not statistically significant.

### Outcomes

Complication rates were 9.1% (three pancreatitis and one cholangitis cases) and 12.8% (four pancreatitis and six cholangitis cases) in the EPLBD + ML and EPLBD groups, respectively. No bleeding or perforation was observed in either of the groups. There were no significant differences in the incidence or types of complications between the two groups.

**FIG. 2.**
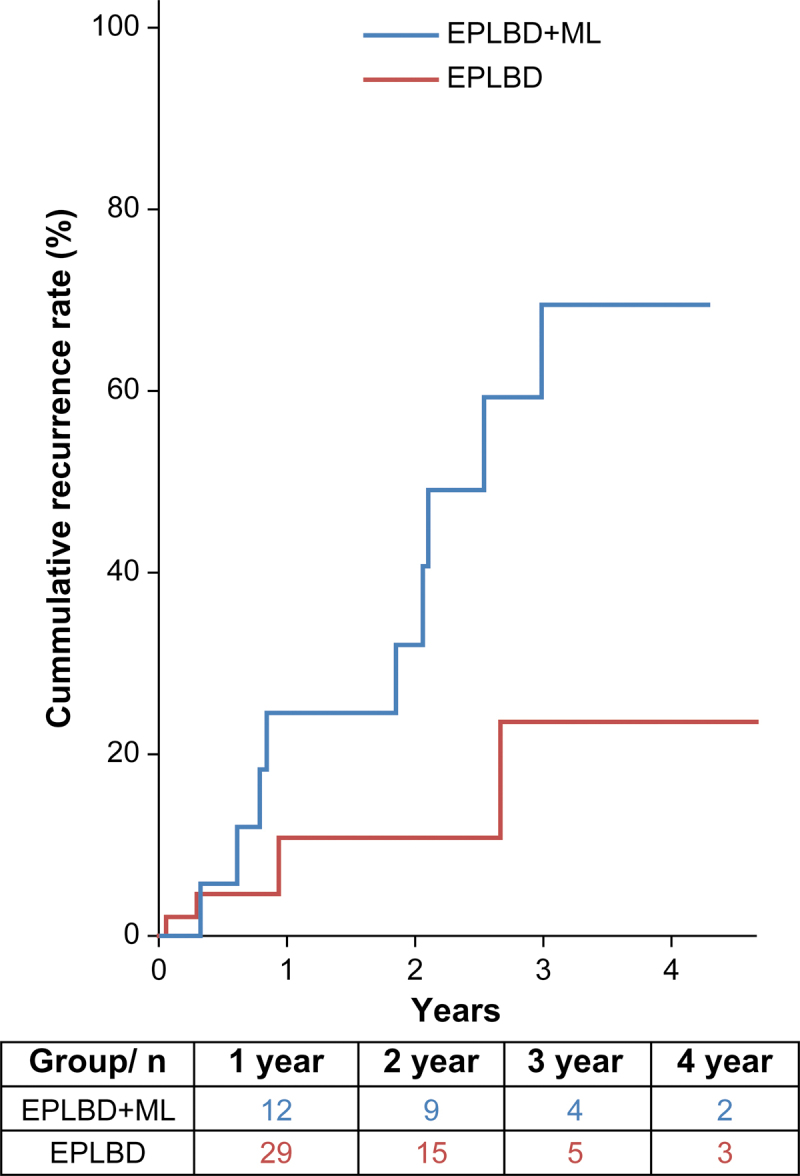
Kaplan–Meier curves showing cumulative recurrence rates in the two study groups.

Cumulative recurrence rates were significantly higher in the EPLBD + ML group (1 year: 24.4%; 2 years: 32.0%; 3 years: 69.4%; and 4 years: 69.4%) than in the EPLBD group (1 year: 10.7%; 2 years: 10.7%; 3 years: 23.5%; and 4 years: 23.5%; *P* = .020) (Fig. 2).

## Discussion

This study aimed to compare the treatment outcomes of EPLBD and EPLBD + ML in patients with difficult common bile duct stones that could not be removed using a balloon or basket catheter during ERCP. Our results showed that ML tended to be added to EPLBD in cases involving older patients with a large maximum stone diameter. Moreover, when ML was added, the number of ERCP procedures performed tended to be two or more, and the total ERCP time tended to be longer. Longer procedure and fluoroscopic times with ML have also been reported in prior studies.^7,8^

Although there were no differences in the rate of complications between the two groups in our study, the long-term recurrence rate was higher in the EPLBD + ML group than in the EPLBD group. Consequently, although it is logical to add ML in cases with large stones that cannot be extracted with EPLBD alone, it is also conceivable that small or fragmented stones may persist after ML and result in a higher long-term recurrence rate.

Furthermore, this study indicates that in addition to large common bile duct stones treatment requiring the addition of ML, it requires a longer total ERCP time and multiple ERCP. In a randomized controlled trial comparing outcomes in patients with large bile duct stones treated with EST+EPLBD versus EST + ML,^9^ postprocedural complications were observed to be more common in the EST + ML group (20% versus 4.4%). These results emphasize the additional burden of performing ML during ERCP. Moreover, our results demonstrate that with appropriate patient selection, it is possible to complete cases easily and successfully remove difficult common bile duct stones using EPLBD without adding ML.

This study has a few limitations. First, it was a single-center retrospective study with a relatively small sample size, and the study groups were inherently different regarding patient and disease characteristics. We did not perform multivariable analysis adjusting for confounders and, therefore, could not ascertain the independent effect of the two treatments on outcomes. Second, we did not standardize the protocols for follow-up and postprocedural imaging in this study. Nonetheless, our results showed that it is possible to complete the treatment of some difficult cases of bile duct stones easily and successfully using EPLBD alone without adding ML. Large multicenter prospective studies are required to confirm whether long-term outcomes differ based on the treatment technique.

## Conclusions

Our results suggest that with appropriate case selection, it is possible to easily and successfully treat difficult biliary stones using EPLBD alone, without the addition of ML.
